# NanoFilter: enhancing phasing performance by utilizing highly consistent INDELs and SNVs in nanopore sequencing

**DOI:** 10.1093/bioinformatics/btaf453

**Published:** 2025-08-13

**Authors:** Shanming Chen, Fan Nie, Jianxin Wang

**Affiliations:** School of Computer Science and Engineering, Central South University, Changsha 410083, China; School of Computer Science and Engineering, Central South University, Changsha 410083, China; School of Computer Science and Engineering, Central South University, Changsha 410083, China

## Abstract

**Motivation:**

Nanopore sequencing data offer longer reads compared to other technologies, which is beneficial for phasing and genome assembly. INDELs provide valuable haplotype information and have significant potential to improve phasing performance. However, accurately identifying INDELs with variant callers is challenging, and incorporating INDELs into phasing remains a complex task. To address these issues, we developed NanoFilter, a novel filtering strategy designed to filter out INDELs that contain wrong phasing information based on their consistency.

**Results:**

Our assessment using Nanopore R10 simplex data shows that filtering out low-consistency INDELs increases their precision from 88.3% to 98.8%, nearly matching the precision of SNVs. In the phasing results of Margin, incorporating these filtered INDELs leads to a 12.77% increase in N50 length and fewer switch errors. Furthermore, we found that SNVs filtered by NanoFilter will enhance assembly performance. When NanoFilter is integrated into the HapDup assembly pipeline, NanoFilter reduces the Hamming error rate and increases N50 length by 7.8%.

**Availability and implementation:**

NanoFilter is available at https://github.com/Chenshanming-repo/NanoFilter (DOI: 10.5281/zenodo.16777826) and HapDup-NanoFilter is available at https://github.com/Chenshanming-repo/HapDup-NanoFilter (DOI: 10.5281/zenodo.16777890).

## 1 Introduction

Phasing is the process of reconstructing haplotypes, which are groups of alleles at variant sites on the same chromosome that are inherited together (Edge *et al.* 2017). In diploid organisms such as humans, each individual has two haplotypes, one inherited from each parent. Reconstructing these haplotypes provides a complete picture of genetic variation in an individual. Understanding haplotypes offers valuable information on various aspects of population genetics, including population structure, migration, and environmental pressures (Martin *et al.* 2023). However, haplotype reconstruction faces several challenges. First, the distribution of variants is uneven, with certain regions exhibiting sparse heterozygous variants, which can complicate accurate phasing and variant calling. To address this, long sequencing reads that can span multiple heterozygous variant sites are required to capture the relationships between them. Second, errors in variant calling and alignment inaccuracies can introduce incorrect phasing information, complicating the haplotype reconstruction process.

Nanopore sequencing offers longer reads compared to short reads and HiFi reads, with short reads averaging ∼100 bp (Kim *et al.* 2021), Single Molecule Real-Time (SMRT) sequencing producing HiFi reads averaging ∼20 kb, and Oxford Nanopore Technologies’ nanopore sequencing generating ultra-long reads averaging ∼100 kb (Marx 2023), enhancing the accuracy and comprehensiveness of variant phasing (Edge *et al.* 2017). However, due to the higher error rate of Nanopore sequencing, which typically ranges from 5% to 15%, the performance of variant callers is often suboptimal, particularly for INDELs (Dunn *et al.* 2023). Clair3 (Zheng *et al.* 2022) and PEPPER (Shafin *et al.* 2021) are among the leading tools for small variant calling in Nanopore reads, demonstrating excellent performance in SNV detection with an F1 score exceeding 99.5%. However, their performance in identifying INDELs is notably worse, with F1 scores around 80%, highlighting the ongoing challenge of reliable INDEL detection.

After variant calling, phasing tools are used to assign variants to their respective haplotypes. WhatsHap is one of the most widely used tools for this purpose, separating long reads into two groups by solving the NP-hard problem of minimum error correction (Martin *et al.* 2023). Margin, which uses a hidden Markov model for haplotype phasing, maintains high accuracy, and contiguity on Nanopore data (Shafin *et al.* 2021). In comparison, HapCUT2 can generate longer phase blocks but introduces more switch errors within those blocks (Edge *et al.* 2017). Although these tools perform well in phasing SNVs alone, our experiments have shown that their performance tends to decline when INDELs are incorporated into the phasing process. Phasing both SNVs and INDELs increases the length of N50, but also results in more switch errors compared to phasing SNVs alone, since errors in INDEL calling and allele assignment can lead to incorrect phasing information for SNVs. INDELs, which represent the second most frequent form of variation in the human genome (Edge *et al.* 2017), offer valuable information for phasing. However, effectively utilizing INDELs to improve phasing performance remains a challenging problem.

To address these issues, we developed NanoFilter, a novel filtering strategy designed to enhance the precision of INDELs on the basis of their consistency. In our experiments on Nanopore sequencing datasets, our method significantly improved the precision of heterozygous INDEL sites after filtering out low-consistency variants. Furthermore, the phasing tools using NanoFilter-filtered INDELs improved the N50 length of the phase blocks while reducing switch errors compared to SNV-only phasing. Finally we integrated NanoFilter into HapDup (Kolmogorov *et al.* 2023), the refined pipeline produced assemblies with longer phased block N50 length and lower Hamming errors rates.

## 2 Materials and methods

### 2.1 Overview

**Figure 1.**
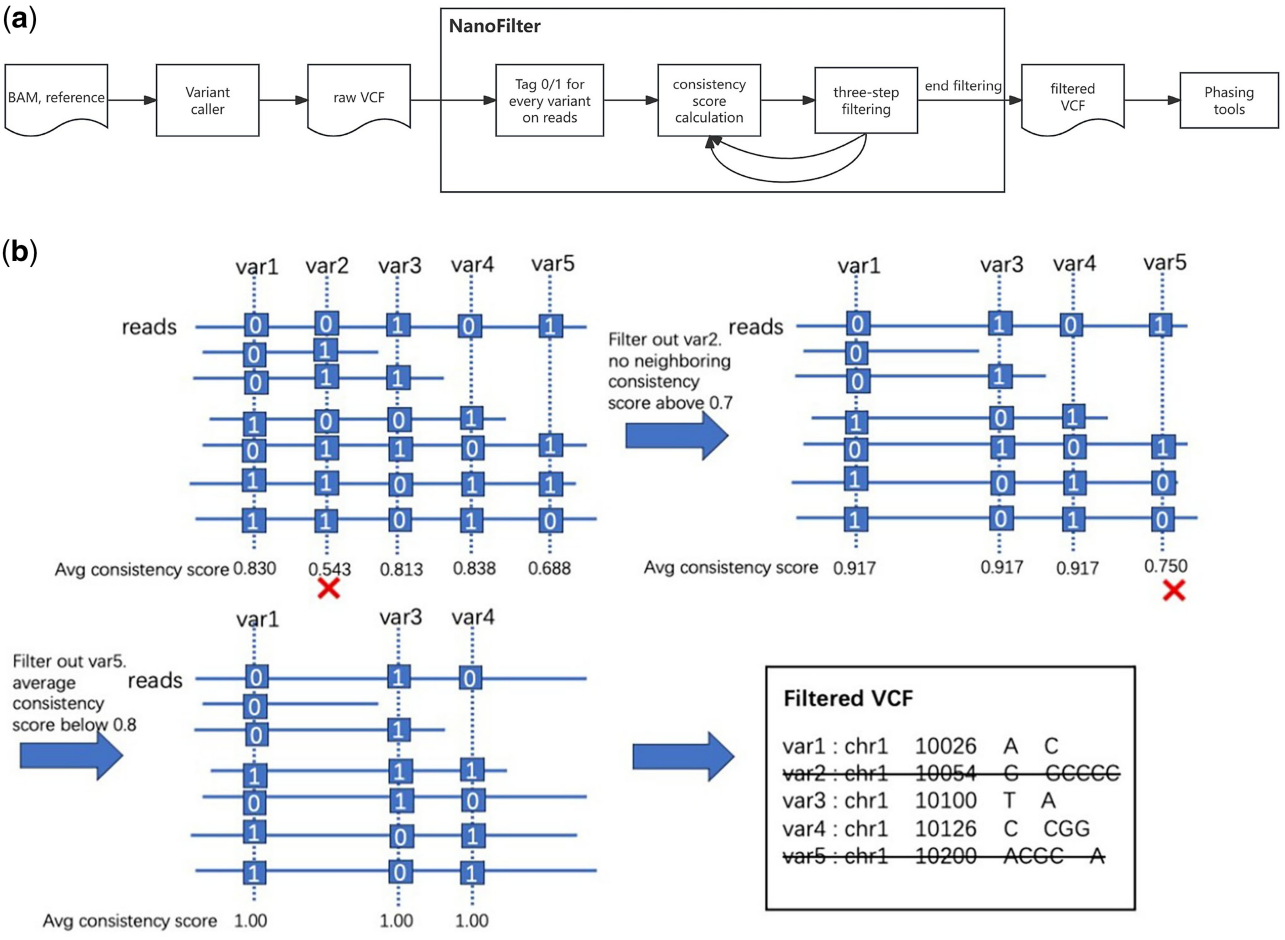
(a) Pipeline of NanoFilter. (b) An example of consistency calculation and filtering strategy in NanoFilter pipeline.

As shown in Fig. 1a, NanoFilter is a tool designed to filter VCF files generated by variant callers. It takes a BAM file and its corresponding VCF file as input, and outputs a filtered VCF file that can be used for downstream phasing by phasing tools. NanoFilter begins with the extractHAIRS command from the HapCUT2 tool, which tags each variant site in the VCF file for every read. A tag of 0 indicates no variant, while a tag of 1 indicates the presence of a variant. Next, NanoFilter computes a consistency score for each pair of variant sites based on their co-occurrence patterns across all reads. This score quantitatively reflects how often two variants are observed on the same haplotype, serving as a proxy for the phasing consistency between them. The underlying assumption is that true heterozygous variants on the same haplotype should exhibit high consistency across supporting reads, whereas false positives or poorly supported sites will show low or inconsistent linkage patterns. Based on these scores, a filtering strategy is applied to remove sites that do not meet the defined criteria. The final output includes the filtered VCF file, along with the average consistency score for each variant site and the specific filtering criteria applied.

### 2.2 Consistency score

In this algorithm, the primary criterion for filtering a variant is the consistency of this variant site with other neighboring INDEL and SNV sites. When describing a variant of parental differences (REF/ALT), we typically use 0 or 1 to indicate whether a variant is present at a particular site. When a pair of variants of parental difference appear together on *N* reads, *x* of those reads will have a combination of 01 or 10, while *y* will have a combination of 00 or 11 (where *x* + *y* = *N*). We refer to these two groups of reads as being consistent with each other at this pair of variants, and the consistency score is defined as follows:


(1) $$\text{consistency-score}=\frac{\max(x,y)}{x+y}.$$

If there are no errors in allele assignment or variant calling, the consistency score reaches its maximum of 1. A higher consistency score indicates that the two variants show more consistent phasing information across the reads that cover them.

### 2.3 Filtering strategy

As illustrated in Fig. 1b, filtering unreliable variant sites is crucial for improving overall consistency scores. In regions with a mix of reliable and unreliable variant sites, the presence of inconsistent sites (such as var2 and var5) can lower the average consistency score of reliable sites. By progressively removing these unreliable sites, the consistency of the remaining variant sites improves, ultimately reaching a consistency score of 1. To achieve this, a stepwise filtering approach is applied.

**Figure 2.**
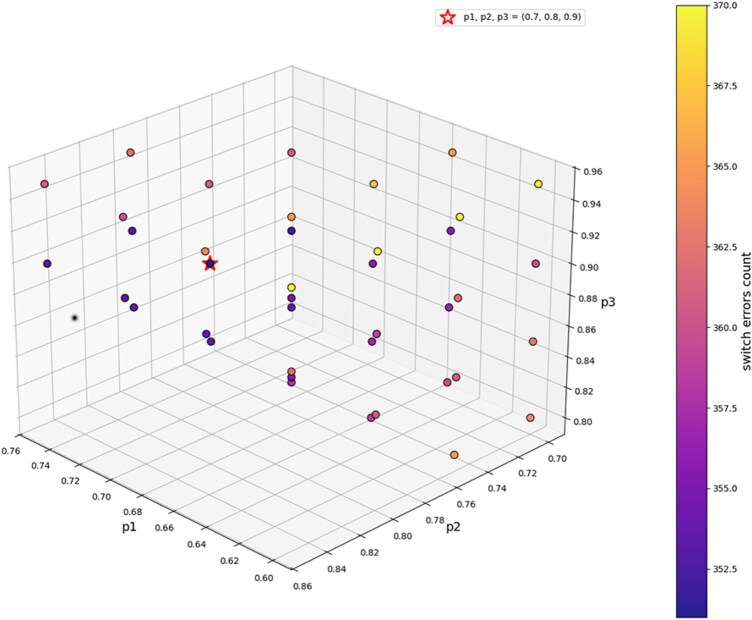
Phasing results of raw SNVs and INDELs filtered by NanoFilter with different filtering parameters on HG002 R10 duplex data.

To determine the optimal filtering parameters for the three-step filtering of NanoFilter, we tested various combinations of $\left(p_{1},p_{2},p_{3}\right)$ thresholds for INDEL filtering, followed by phasing with Margin on HG002 R10 duplex data. Here, $p_{1}$, $p_{2}$, and $p_{3}$ correspond to the first, second, and third filtering thresholds applied in the three-step filtering strategy, respectively. The tested parameter ranges were $p_{1}\in \{0.6,\;0.65,\;0.7,\;0.75\}$, $p_{2}\in \{0.7,\;0.75,\;0.8,\;0.85\}$, and $p_{3}\in \{0.8,\;0.85,\;0.9,\;0.95\}$, with the constraint that $p_{1}<p_{2}<p_{3}$. As shown in Table 1, available as supplementary data at *Bioinformatics* online, all combinations yield similar N50 values, indicating stable phasing block lengths. However, the combination (0.7, 0.8, 0.9) results in the lowest switch errors count, and is therefore selected as the default parameter set for the three-step filtering process. A full visualization of all parameter combinations is provided in Fig. 2. Notably, overly stringent filtering parameter (e.g. $p_{3}=0.95$) leads to increased switch errors, likely due to the loss of informative variants. In contrast, increasing the stringency of $p_{1}$ and $p_{2}$ generally lead to a clearer reduction in switch errors, highlighting the importance of first and second filtering step in optimizing INDEL filtering for phasing performance.

**Table 1.** Summary of sequencing data used in this study.

Data	Coverage (fold)	Accuracy (%)	N50 (bp)	N90 (bp)
HG002 R9	87	94.1	29 612	10 026
HG002 R10 simplex	47	97.4	29 803	9928
HG002 R10 duplex	30	95.7	39 135	15 543
HG001 R9	60	97.6	25 806	8801

Based on the selected optimal parameters $\left(p_{1},p_{2},p_{3})=(0.7,0.8,0.9\right)$, the three-step filtering process begins by removing sites with no neighboring consistency score above 0.7, as these sites are unlikely to contribute to reliable phasing. Next, sites with an average consistency score below 0.8 are filtered out. Finally, a stricter threshold of 0.9 is applied to remove any remaining low-consistency sites. In regions with insufficient coverage, where reliable consistency scores cannot be calculated, variant quality scores from the VCF file are used as an additional filtering criterion, with a default threshold of quality 15.

### 2.4 Integration of HapDup

HapDup is a pipeline designed to convert a haploid assembly into a diploid assembly. Initially, Flye is used to assemble long reads into a haploid reference sequence. The reads are then realigned to the reference to produce an alignment file. PEPPER is subsequently used to call SNVs and INDELs from the alignment file. After variant calling, NanoFilter is applied to filter the variants, improving their consistency and quality before they are passed to the phasing step. Margin is then used to phase the filtered variants and assign reads to their respective haplotypes. Once the reads are phased, Flye is applied again to polish the initial assembly for each haplotype. The original HapDup pipeline does not use INDELs for phasing. To enhance this, we have integrated NanoFilter into the pipeline. This integration allows HapDup to incorporate both high-consistency SNVs and INDELs for phasing, resulting in improved assembly performance. We have also validated the effectiveness of NanoFilter through this enhanced pipeline.

### 2.5 Benchmark methods

For benchmarking filtering and phasing performance, we used publicly available Genome in a Bottle phased variant set v4.2.1 (GRCh38) for the HG001 and HG002 data, which include haplotype information for both SNVs and INDELs.

In the GIAB benchmark, SNVs and INDELs are called using PEPPER and evaluated using hap.py, a suite of tools built on htslib for benchmarking variant calls against gold-standard truth datasets, with precision and recall as the primary evaluation metrics. Variants obtained after applying different filtering strategies are also assessed using hap.py to ensure consistent comparisons.

In the phasing results benchmark, SNVs and INDELs called by PEPPER are used as the raw variant sets. Two additional INDEL variant sets are generated using two different filtering strategies: NanoFilter with default parameters and a quality-based filtering method. The quality-based filtering method is implemented by out custom script which uses Quality column in VCF file to filter INDELs. The two filtering methods result in two different filtered INDEL sets: INDELs filtered by NanoFilter and INDELs filtered by the quality-based filtering method. We then use WhatsHap to calculate and compare the N50 block length and the number of switch errors for the phasing outputs. Notably, only SNVs are used as the benchmark set during phasing evaluation, as they are not subject to filtering. This ensures that the variant set remains consistent across all phasing comparisons.

After HapDup outputs two assemblies, we evaluate them using Merqury (Rhie *et al.* 2020). Merqury is a benchmark tool for evaluating genome assemblies with k-mers. For benchmarking HapDup performance, we focus on phased block N50 length and hamming error rate of two assemblies calculated by Merqury benchmark results.

### 2.6 Datasets and preprocessing

In this study, we conducted experiments using four Nanopore datasets. Detailed information can be found in Table 1. For the experiments in the HapDup benchmark, we first assemble the reads from the BAM file into a single haplotype using Flye. Then, these reads are realigned to the newly assembled haplotype by Minimap2 to generate a SAM file, using the haplotype sequence as the reference sequence in the HapDup pipeline. Finally, we use Samtools to convert the SAM file to a BAM file and generate an index file.

## 3 Results

### 3.1 GIAB variant set benchmark

When using variant callers for SNV and INDEL detection, we typically evaluate the performance of the variant caller using the hap.py tool with the GIAB variant set used as the gold standard, showcasing the precision and recall of the detected variants. Here, we used PEPPER to call the SNV and INDEL sites across the entire genome and then used hap.py to benchmark the precision of the raw SNVs and INDELs. After filtering out some INDEL sites by NanoFilter, we can similarly evaluate the quality and filtering performance of the processed INDELs to assess the impact of NanoFilter. Since the phasing tools only utilize variants with a heterozygous genotype of 0/1, we have selected the sites with a heterozygous genotype of 0/1 from the GIAB gold standard set as the evaluation benchmark.

**Figure 3.**
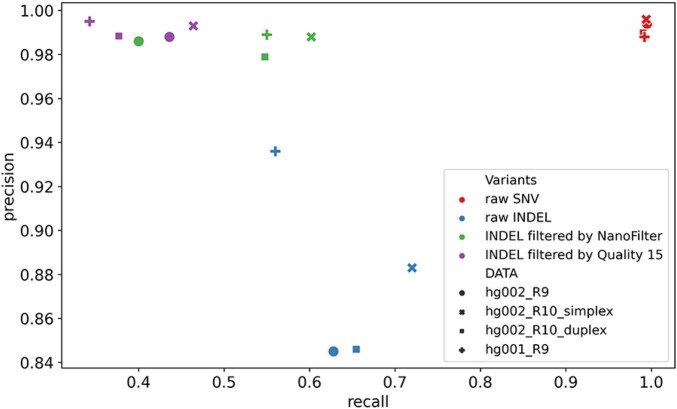
Precision and recall of different SNV and INDEL variant sets evaluated using the GIAB benchmark set.

To facilitate description, we present the benchmark metrics for the four datasets in the format (x, x, x, x), where each value corresponds to a dataset as summarized in Table 1. Figure 3 shows a consistent trend in all data, where the raw INDEL sites have lower precision and recall compared to raw SNV sites. However, by sacrificing a small amount of recall, INDEL precision can be improved to closely match that of SNVs. After filtering low-consistency INDEL sites, the precision of the INDELs filtered by NanoFilter increases from (84.5%, 88.3%, 84.6%, 93.6%) to (98.6%, 98.8%, 97.9%, 98.9%), nearly approaching the precision of raw SNV sites, which is (99.4%, 99.6%, 99.0%, 98.8%). As a consequence, the recall of INDEL sites filtered by NanoFilter decreases from (62.8%, 70.2%, 65.4%, 56.0%) to (40.0%, 60.2%, 54.7%, 55.0%).

To further explore the impact of different INDEL filtering strategy on variant calling optimization, we also evaluated the INDEL sites filtered by a fixed quality threshold of 15, based on the QUAL field in the VCF file. This approach represents a simple filtering method, where only high-quality variants, as defined by the variant caller, are retained. As shown in Fig. 3, the INDELs filtered by Quality 15 yield a precision of (98.8%, 99.3%, 98.8%, 99.5%), which is comparable to the precision of the INDELs filtered by NanoFilter. However, the INDELs filtered by a fixed quality threshold 15 obtain a recall of (43.6%, 46.4%, 37.7%, 34.3%), generally below the recall achieved by NanoFilter, which is (40.0%, 60.2%, 54.7%, 55.0%). Although NanoFilter recall is slightly lower in one dataset, it substantially outperforms quality-based filtering in the other datasets, demonstrating a better overall balance between precision and recall.

In summary, while both quality-based filtering and NanoFilter significantly enhance INDEL precision, NanoFilter exhibits a superior trade-off between precision and recall. By leveraging consistency information from sequencing reads rather than relying solely on quality scores, NanoFilter retains more true heterozygous INDEL sites without compromising much precision. This makes it a more effective strategy for improving INDEL quality prior to downstream phasing and haplotype-aware analyses.

### 3.2 Phasing results

In this section, we evaluated the phasing performance using three different phasing tools: Margin, WhatsHap, and HapCUT2, to phase different sets of variants. We generated VCF files by applying quality-based INDEL filtering using our custom script, leveraging variant caller quality scores. These filtered variant sets were then compared against NanoFilter to assess their respective impacts on phasing performance. To quantify phasing performance, we used WhatsHap stats and WhatsHap compare to evaluate the phased VCF against the GIAB truth set. Notably, we benchmarked only SNVs in the phased VCF, as the SNV set remained unchanged across all phasing experiments, ensuring consistency.

**Figure 4.**
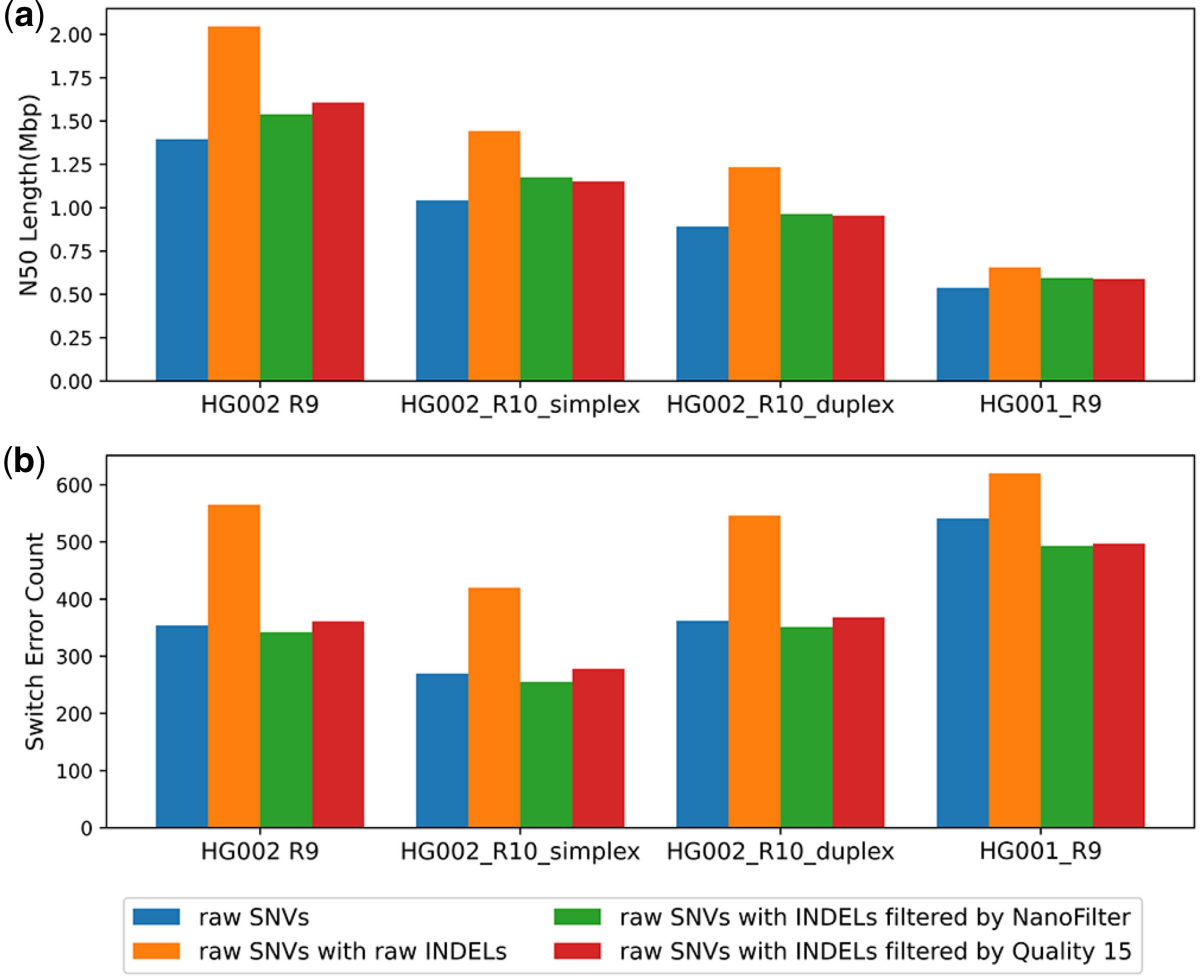
Haplotype-phasing performance assessed with Margin across datasets and variant-call sets: (a) N50 contig length; (b) Number of switch error.

As shown in Fig. 4, compared to phasing with raw SNVs alone, including raw INDELs into phasing with Margin increases the N50 length but also raises switch errors count from (354, 270, 320, 473) to (565, 419, 362, 541). Incorporating INDELs filtered by NanoFilter into phasing improves the N50 length by percentage of (10.35%, 12.77%, 8.03%, 10.83%) compared to phasing with raw SNVs alone and reduces switch errors count to (342, 255, 303, 459) which is lower than that of phasing results of raw SNVs. Compared to phasing with raw SNVs alone, incorporating INDELs filtered by NanoFilter into phasing produces longer N50 length and fewer switch errors. This demonstrates that the INDELs filtered by NanoFilter effectively optimize the phasing performance of SNVs, serving as a valuable complement to SNVs. Additionally, across all datasets, using INDELs filtered by NanoFilter results in fewer switch errors than using INDELs filtered with a quality threshold of 15, which yields switch errors count of (361, 278, 368, 497). In all datasets except HG002 R9, using INDELs filtered by NanoFilter leads to a higher N50 length compared to those filtered by quality 15. In HG002 R9, however, using NanoFilter-filtered INDELs results in a slightly lower N50 length, with percentage of 4.35%.

**Figure 5.**
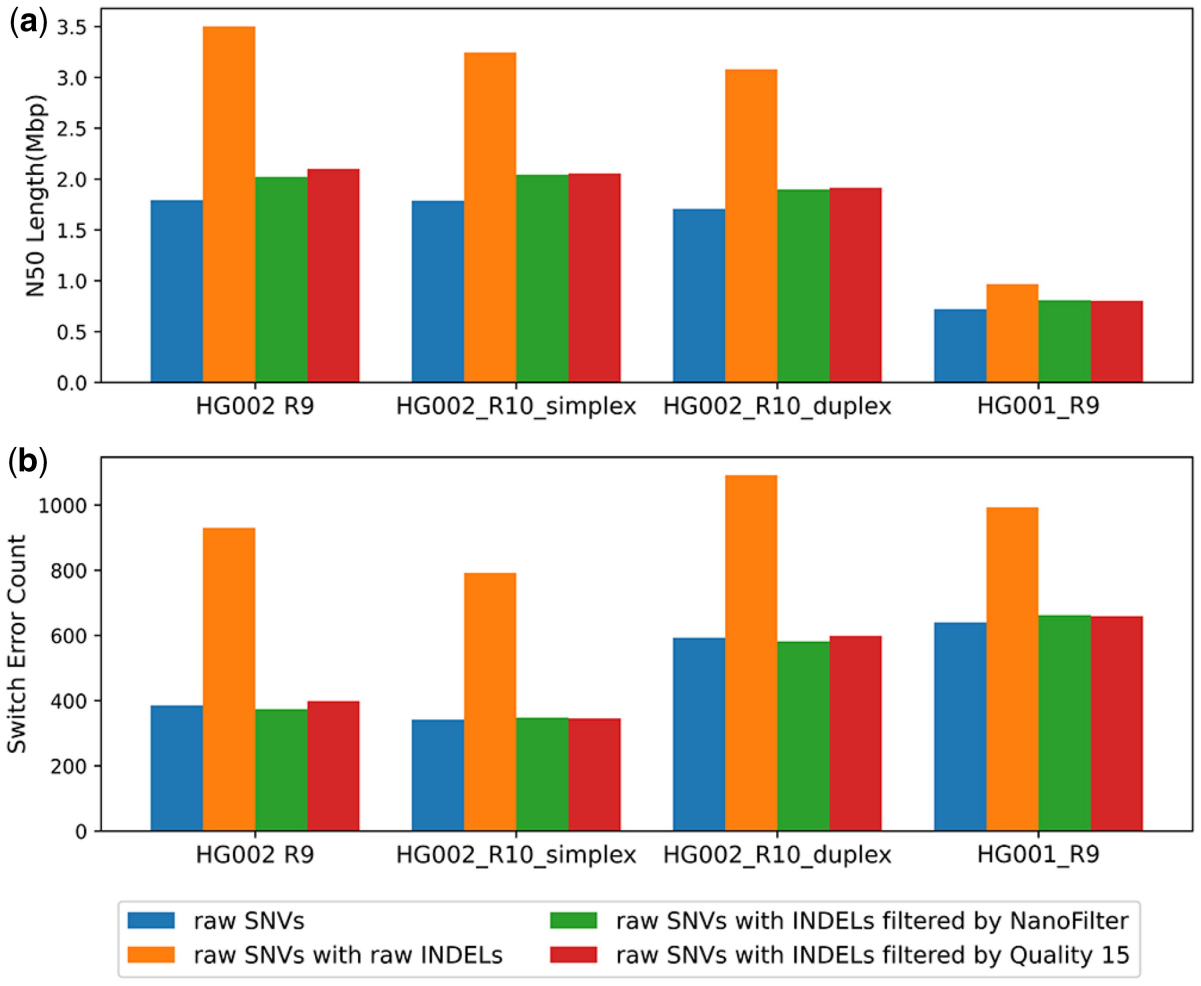
Performance of haplotype phasing assessed with WhatsHap across input datasets and variant-call sets. (a) N50 contig length. (b) Number of switch errors.

As shown in Fig. 5, compared to phasing with raw SNVs alone, including raw INDELs into phasing with WhatsHap increases the N50 length but also raises switch errors count from (385, 342, 593, 640) to (930, 792, 1092, 993). After using INDELs filtered by NanoFilter, switch errors count change to (374, 348, 582, 662) while the N50 length increases with percentage of (15.72%, 14.90%, 11.72%, 11.87%) compared to phasing with raw SNVs alone. Compared to using raw INDELs, phasing with INDELs filtered by NanoFilter reduces switch error levels to those of phasing results of raw SNVs, while also improving the N50 length to a certain extent. For switch errors count, using INDELs filtered by NanoFilter results in fewer switch errors in HG002 R9 and HG002 R10 duplex data compared to using INDELs filtered by Quality 15, with reductions of 25 and 17 respectively. However, in the other two datasets, using INDELs filtered by NanoFilter leads to a slight increase in switch errors count compared to using INDELs filtered by Quality 15, with increases of 2 and 3 respectively. Regarding N50 length, filtering with Quality 15 provides a 3.92% increase in HG002 R9, while for the other data, the N50 length are comparable.

**Figure 6.**
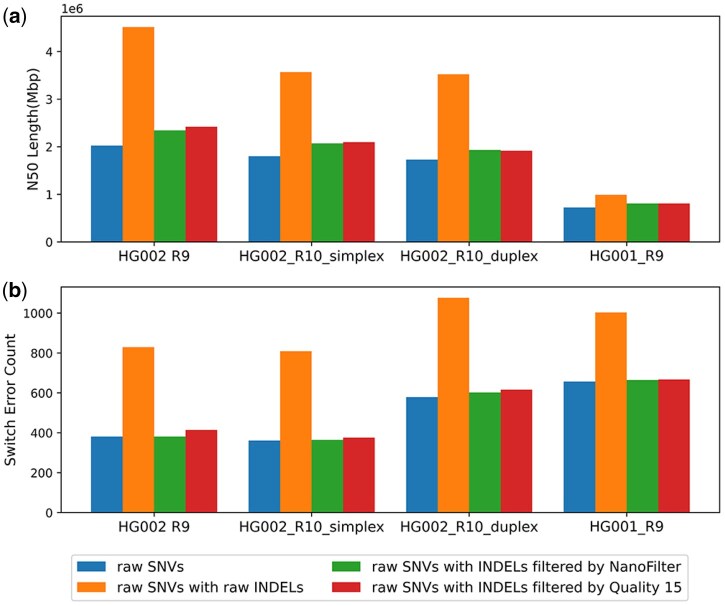
Performance of haplotype phasing assessed with HapCUT2 across input datasets and variant-call sets. (a) N50 contig length. (b) Number of switch errors.

As shown in Fig. 6, compared to phasing with raw SNVs alone, including raw INDELs into phasing with HapCUT2 increases the N50 length but also raises switch errors count from (381, 361, 579, 657) to (829, 809, 1077, 1003). After filtering INDELs with NanoFilter, switch errors count decreases into (381, 364, 602, 665) while the N50 length increases with percentage of (12.66%, 14.28%, 11.26%, 11.99%) compared to phasing with raw SNVs alone. Compared to raw INDELs, using NanoFilter-filtered INDELs for phasing brings switch errors count down to those of phasing results of raw SNVs. Across all data, INDELs filtered by NanoFilter consistently show less switch errors count than those filtered by Quality 15. In terms of N50 length, NanoFilter-filtered INDELs result in a decrease compared to Quality 15 filtering in the HG002 R9 and HG002 R10 simplex datasets, with percentage of 3.20% and 1.28%, respectively. While for the other 2 data, the N50 length are comparable.

In summary, incorporating filtered INDELs consistently improves N50 length across all tools compared to phasing with raw SNVs. For Margin, it also reduces switch errors count compared to phasing with raw SNVs. For WhatsHap and HapCUT2, while filtered INDELs does not always reduce switch errors compared to phasing with raw SNVs, they help maintain switch errors count at levels comparable to using raw SNVs alone. Overall, the N50 length of phasing results using simple quality filtering is comparable to those obtained with NanoFilter. However, the switch errors are typically higher with quality-based filtering.

**Figure 7.**
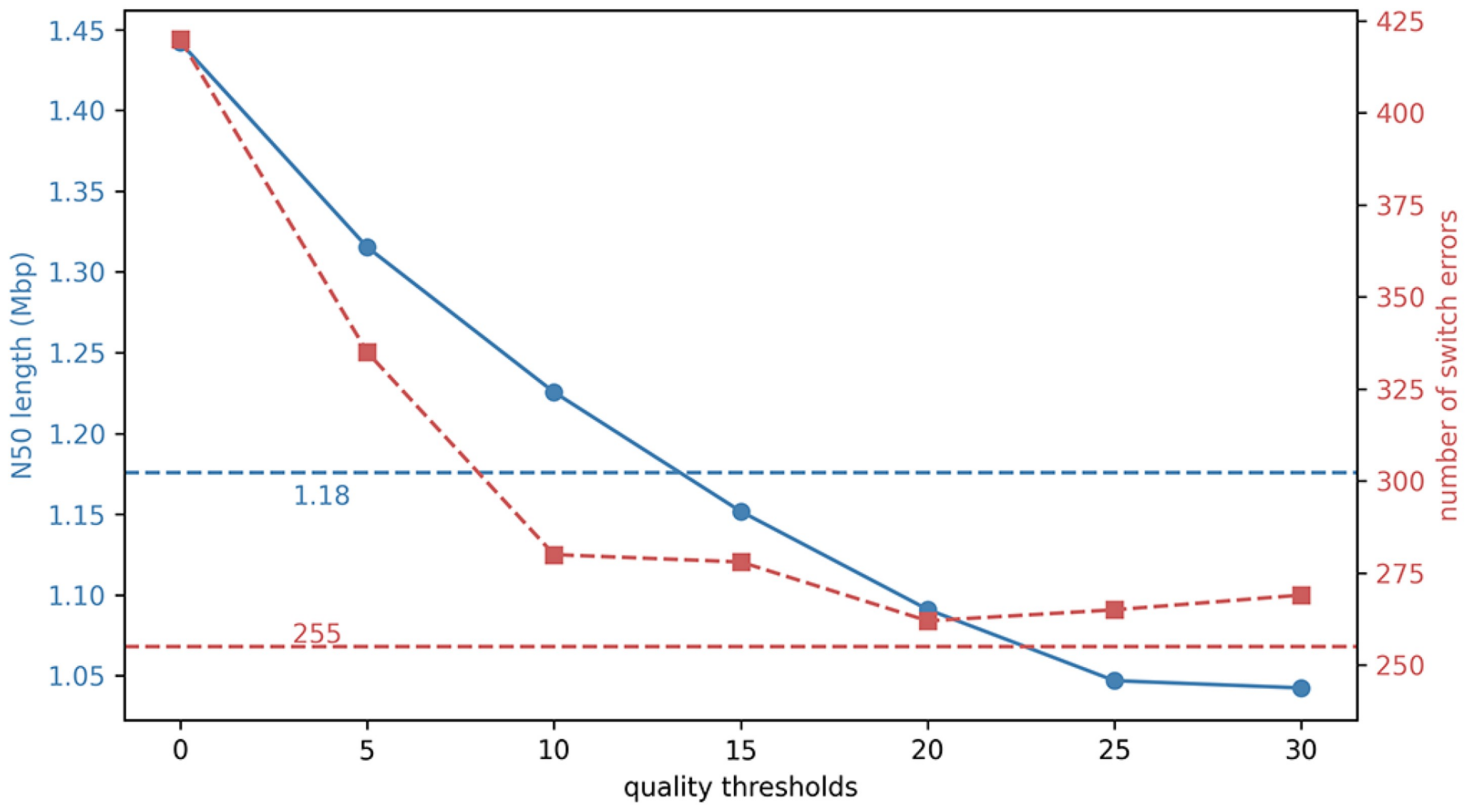
Phasing performance on HG002 R10 simplex with different INDEL filtering strategies. Horizontal dashed lines indicate results from using NanoFilter with default parameters (N50 = 1.18 Mbp, switch errors = 255).

In addition, we also compared NanoFilter with a series of quality-based INDEL filtering thresholds to explore their effects on phasing. As shown in Fig. 7, the Margin phasing results on the HG002 R10 simplex dataset demonstrate that combining raw SNVs with INDELs filtered by NanoFilter achieves the lowest switch errors count (255) while maintaining a relatively high N50 length (1.18 Mbp). In contrast, applying quality-based INDEL filtering alone—ranging from quality threshold of 0 to 30 fails to deliver comparable improvements. As the quality threshold increases from 0 to 30, the N50 length steadily declines from 1.23 to 1.04 Mbp, suggesting that overly stringent thresholds can eliminate informative variants crucial for constructing long haplotypes. Meanwhile, the switch error does not exhibit a consistent downward trend, indicating that raising the quality cutoff does not reliably improve phasing accuracy.

Interestingly, a moderate threshold of 20 does lead to a reduction in switch errors count, likely due to the removal of some false-positive INDELs. However, the switch errors still remain higher than that achieved using NanoFilter. As the threshold increases further, more true-positive INDELs are also filtered out, resulting in the loss of valuable phasing information and a corresponding rise in switch errors. These findings highlight the limitations of using a fixed quality threshold for INDEL filtering. In contrast, NanoFilter provides a more adaptive strategy, selectively removing low-consistency variants while preserving those that contribute to accurate and continuous haplotype reconstruction.

### 3.3 Haplotagging accuracy evaluation

We used the GIAB Truth Phased VCF for haplotagging accuracy evaluation, and applied WhatsHap haplotagging on the HG002 R10 simplex BAM as the ground truth for read haplotypes. For testing, we focused on chromosome 20 of HG002, using four different variant sets: (i) SNVs only, (ii) SNVs with raw INDELs, (iii) SNVs with INDELs filtered by NanoFilter, (iv) SNVs with INDELs filtered by quality 15. We performed margin phasing and whatsHap haplotagging, and displayed the haplotagging accuracy distribution and the average haplotagging accuracy for each variant set in Fig. 1, available as supplementary data at *Bioinformatics* online (panels a, b, c, d), with results divided into 1 kb windows. As shown in the figure, the use of INDELs filtered by NanoFilter resulted in a higher average haplotagging accuracy compared to using raw INDELs and quality-filtered INDELs. And the haplotagging accuracy for variant set C was comparable to that of variant set A. Moreover, we observed that the percentage of reads not haplotagged using raw SNVs was 19.44%, which was higher than the percentages for variant set B (17.61%), variant set C (18.73%), and variant set D (18.79%). These results suggest that the use of filtered INDELs (either by quality or NanoFilter) in combination with SNVs leads to improved haplotagging performance on percentage of reads not haplotagged. However, INDELs filtered by NanoFilter achieve even better haplotagging accuracy than that of using quality-based filtering methods, as NanoFilter more effectively retain informative variants while removing erroneous ones.

### 3.4 Assembly results

As shown in Table 2, for the phased block N50 length, the assembly results of all four data follow a similar trend: using variant set B results in the longest N50 length, followed by variant set C and variant set D, while variant set A yields the shortest phased block N50 length. This clearly indicates that using fewer variants leads to shorter phased block N50 lengths. Although variant set B achieves the longest phased block N50 length, it also introduces the highest Hamming error rate. However, when filtered variant sites are used for phasing and assembly, the Hamming error rates are significantly reduced. Notably, compared to the results of variant set A, simultaneously filtering SNV and INDEL sites with NanoFilter still leads to an increase in N50 length by percentage of (3.1%, 7.8%, 4.7%, and 3.7%), which can be attributed to the inclusion of high-confidence INDEL sites. At the same time, the hamming error rate decreases from (1.01%, 0.65%, 0.77%, and 1.96%) to (0.90%, 0.59%, 0.54%, and 0.99%) when compared to the original HapDup pipeline which only utilizes variant set A for phasing and assembly.

**Table 2.** Assembly results benchmarked by Merqury.^a^

Data	Variant set	Size (Gb)	Phase block N50 (Mb)	Switch error rate (%)	Hamming error rate (%)
HG002 R9	A	2.50/2.50	1.39/1.38	0.140/0.143	1.15/0.87
	B	2.60/2.60	2.21/2.26	0.147/0.145	8.17/7.91
	C	2.51/2.52	1.55/1.57	0.143/0.138	1.58/1.14
	D	2.51/2.51	1.42/1.43	0.154/0.154	0.92/0.87
HG002 R10 simplex	A	2.51/2.51	1.16/1.17	0.047/0.051	0.58/0.71
	B	2.57/2.57	1.63/1.64	0.049/0.052	1.58/1.38
	C	2.53/2.54	1.33/1.34	0.048/0.053	0.59/0.62
	D	2.53/2.54	1.26/1.25	0.054/0.055	0.56/0.61
HG002 R10 duplex	A	2.46/2.46	0.92/0.93	0.093/0.099	0.66/0.88
	B	2.52/2.53	1.31/1.29	0.095/0.097	1.75/1.88
	C	2.48/2.49	1.07/1.08	0.095/0.099	0.81/0.86
	D	2.47/2.48	0.96/0.98	0.098/0.099	0.47/0.61
HG001 R9	A	2.19/2.19	0.68/0.67	0.163/0.173	1.83/2.08
	B	2.24/2.24	0.81/0.81	0.172/0.172	2.44/2.25
	C	2.23/2.22	0.76/0.75	0.169/0.174	1.84/1.85
	D	2.19/2.20	0.70/0.70	0.164/0.176	1.02/0.96

a Variant sets A, B, C, and D indicate (a) raw SNVs, (b) raw SNVs with raw INDELs, (c) raw SNVs with filtered INDELs, and (d) filtered SNVs with filtered INDELs, respectively. In the assembly results, two assemblies are generated, representing the paternal and maternal haplotypes. Each column displays two values, corresponding to the respective metrics for each haplotype assembly.

To better understand which genomic regions contain gaps or misassemblies in the assembly results, we used QUAST (Gurevich *et al.* 2013) to align the HapDup-NanoFilter assemblies to the HG002 reference. Specifically, QUAST was used to calculate NGA50 and NGA90 values, which account for misassemblies by breaking contigs at error positions, providing a more accurate measure of effective contiguity and genome correctness. As shown in Table 3, assemblies generated using raw SNVs combined with NanoFilter-filtered INDELs consistently achieve higher NGA50 compared to assemblies using only raw SNVs. Additionally, they exhibit fewer misassemblies in both R10 datasets. These results provide direct evidence supporting the value of incorporating high-consistency INDELs, particularly on R10 datasets where assemblies with filtered INDELs show markedly better contiguity and less assembly errors compared to those using only SNVs. Assembly results of filtered SNVs with filtered INDELs achieves the highest NGA90 values across all assemblies. NGA90 represents the length such that 90% of the reference genome is covered by aligned blocks of at least that length, after breaking contigs at misassembly points. A higher NGA90 indicates a better assembly performance on relatively shorter contigs.

**Table 3.** Assembly results benchmarked by Quast with HG002 reference.^a^

Data	Variant set	NGA50 (Mb)	NGA90 (Mb)	Misassemblies
HG002 R9	A	1.33	0.123	907
	B	2.86	0.121	1193
	C	1.54	0.113	937
	D	1.47	0.137	922
HG002 R10 simplex	A	1.06	0.129	2365
	B	1.51	0.103	2415
	C	1.27	0.124	2357
	D	1.21	0.138	2412
HG002 R10 duplex	A	0.91	0.095	2487
	B	1.23	0.091	2520
	C	1.02	0.095	2472
	D	0.89	0.116	2505

a Variant sets A, B, C, and D indicate (a) raw SNVs, (b) raw SNVs with raw INDELs, (c) raw SNVs with filtered INDELs, and (d) filtered SNVs with filtered INDELs, respectively.

### 3.5 Case study

**Figure 8.**
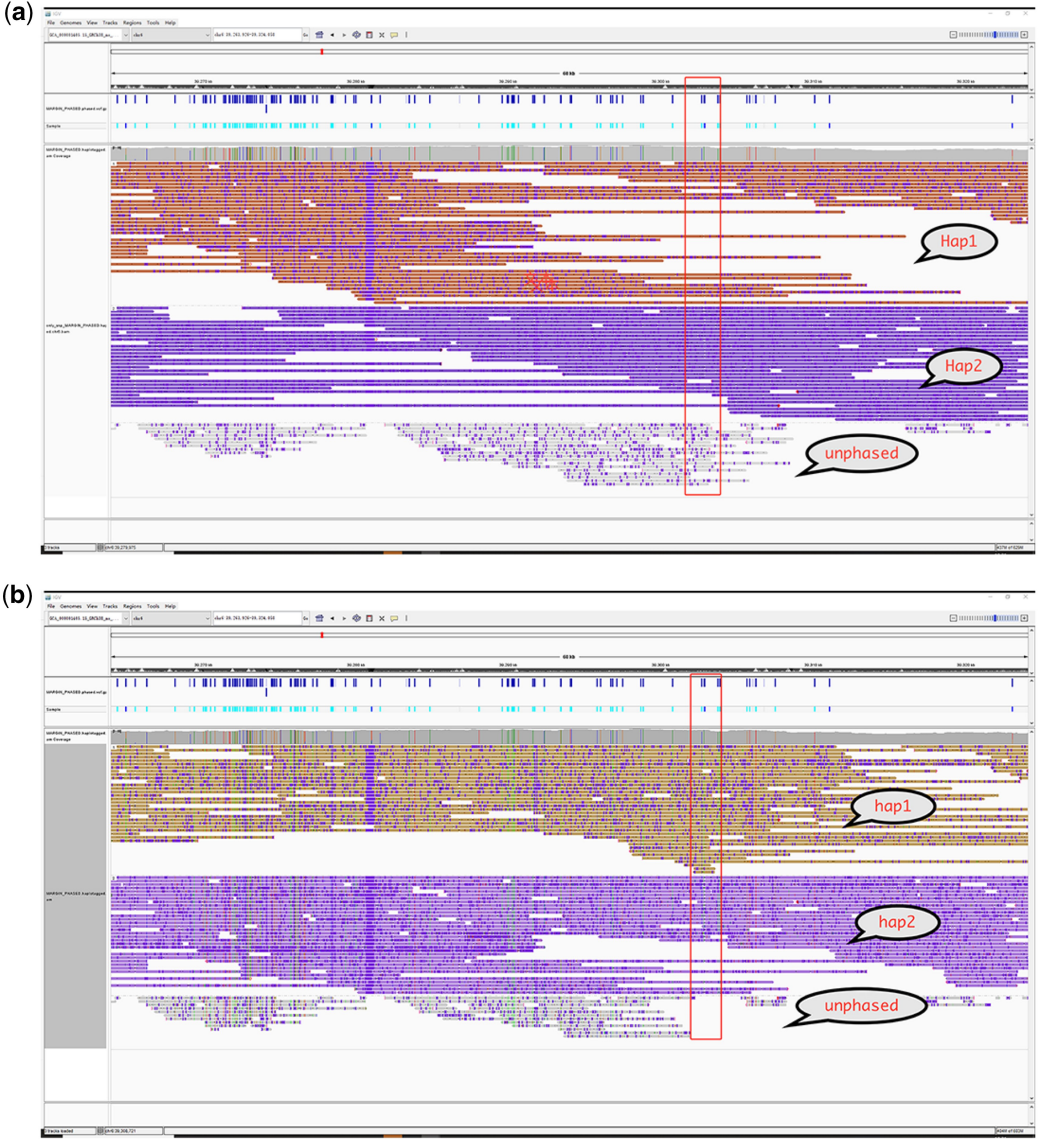
IGV visualization of BAM file haplotagged by different variant sets. (a) IGV visualization of BAM file haplotagged by only SNVs. (b) IGV visualization of BAM file haplotagged by SNVs and INDELs filtered by NanoFilter.

As shown in Fig. 8a and b, IGV displays BAM files haplotagged by raw SNVs and raw SNVs with INDELs filtered by NanoFilter, respectively. In this particular region, no heterozygous SNVs are available for phasing, but a single heterozygous INDEL enables haplotype assignment. Incorporating INDELs allows all reads to be phased, and a switch error within this region is also corrected. While INDELs can serve as a valuable supplement for SNV-based phasing, but some INDEL sites may also introduce errors and lead to a significant increase in switch errors. NanoFilter effectively balances N50 length and switch error count of phase blocks.

**Figure 9.**
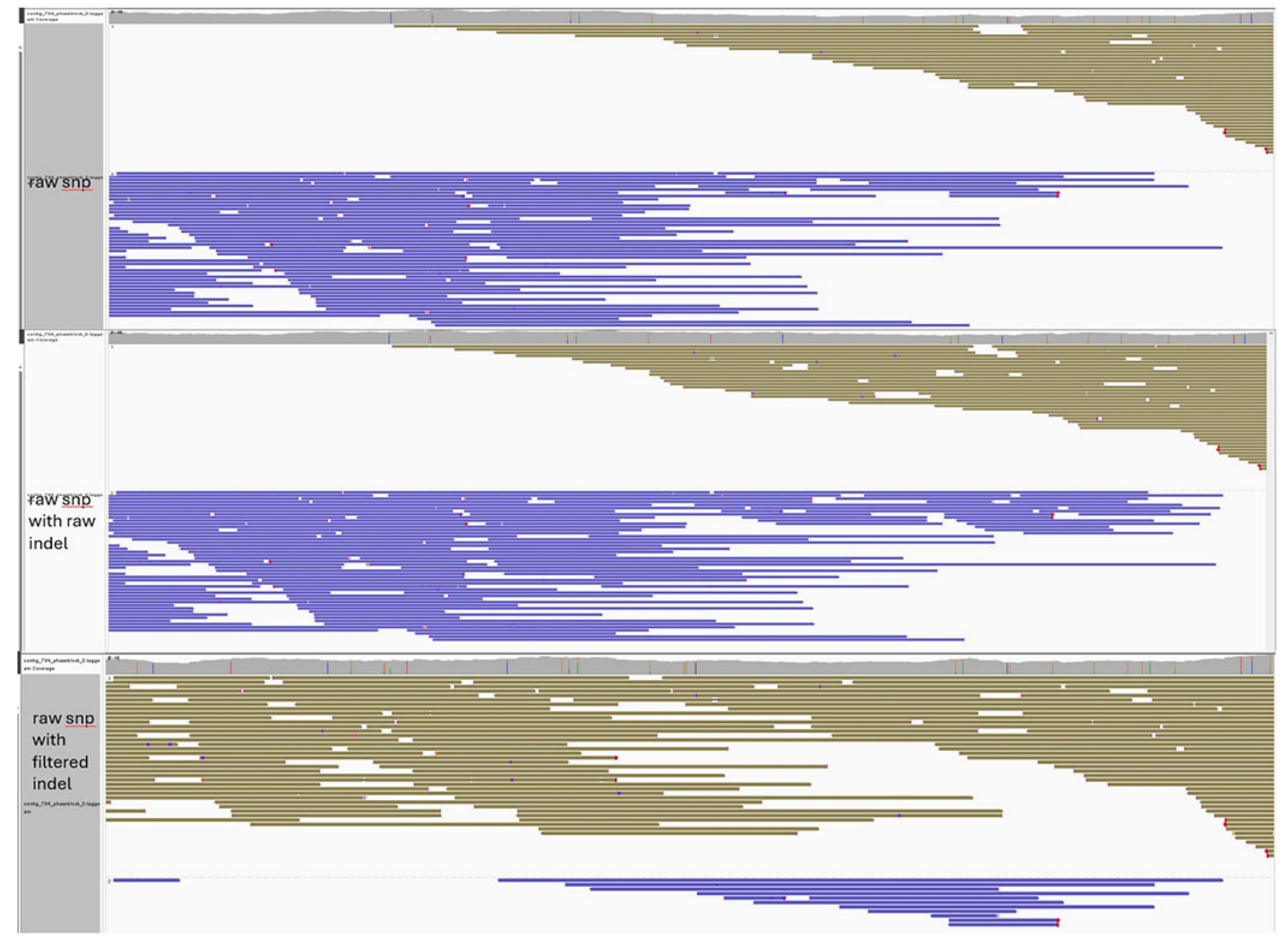
IGV snapshot of read alignments on maternal chromosome 15 (79.28–81.05 Mbp). In each panel, maternal reads are shown above and paternal reads below.

Figure 9 shows that misassigned reads and a haplotype switch when raw SNVs and raw INDELs are used. After filtering low-consistency INDELs, the correct maternal haplotype is restored, improving phasing performance and assembly contiguity.

## 4 Discussion

The application of NanoFilter significantly enhances the performance of phasing tools and the HapDup pipeline. By filtering INDEL variants called from Nanopore data, NanoFilter improves INDEL precision to levels comparable to SNVs, addressing the issue of incorrect phasing information caused by wrong INDEL sites. By filtering INDELs called from Nanopore data, we observed improvements in both N50 length and the number of switch errors, which are critical metrics for phasing. In our study, Margin, which utilized filtered variants, produced more consistent and accurate haplotypes. Similarly, WhatsHap and HapCUT2 also showed enhanced N50 length after filtering, while maintaining number of switch errors comparable to phasing raw SNVs alone. Moreover, our method outperforms simple quality filtering. While the N50 length of the phasing results using quality filtering is comparable to those filtered with NanoFilter, the switch error count is generally higher in the quality-filtered results. These results highlight NanoFilter as a valuable VCF preprocessing step, ensuring greater reliability in haplotype phasing across different tools. Additionally, integrating NanoFilter into the HapDup pipeline prior to assembly results in longer phased block N50 lengths and lower Hamming error rates, improving the overall quality of the assembly. NanoFilter operates by filtering out unreliable variants based on a consistency score threshold, ensuring more accurate variant calls for phasing. Although currently optimized for human genomes, future work will explore extending this method to polyploid organisms, offering potential for broader applications in genomic research.

## Supplementary Material

btaf453_Supplementary_Data

## Data Availability

The NanoFilter tool is available on GitHub at the following repository: https://github.com/Chenshanming-repo/NanoFilter. The HapDup assembly workflow integrated with NanoFilter can be accessed at https://github.com/Chenshanming-repo/HapDup-NanoFilter. Details and links to all sequencing datasets, tool versions, and commands used in this study in the Supplementary Material.
