# Supplementary material for: NanoFilter: enhancing phasing performance by utilizing highly consistent INDELs and SNVs in nanopore sequencing
Source: Bioinformatics. 2025 Aug 13;41(9):btaf453. doi: 10.1093/bioinformatics/btaf453 (PMC12448842; doi:10.1093/bioinformatics/btaf453)
Supplement: btaf453_Supplementary_Data [file btaf453_supplementary_data.pdf]

Supplementary Information

1 Commands

1. Read alignment using Minimap2(v2.24-r1122)

```
flye-minimap2 -ax map-ont -t $THREADS $REF $DATA_DIR/fastq_dir/*.fastq > $DATA_DIR/all.sam
samtools sort -@ $THREADS -m 4G $DATA_DIR/all.sam > $DATA_DIR/all.bam
samtools index -@ $THREADS $DATA_DIR/all.bam
```

2. BAM subsampling using Samtools(v1.21)

```
samtools view -@ $THREADS -b -s 0.$FRAC $DATA_DIR/all.bam > $DATA_DIR/downsample/all_sub.bam
samtools index -@ 40 $DATA_DIR/downsample/all_sub.bam
```

3.PEPPER model

R9 Model

R10 Model

4.Running PEPPER(v0.7.5)

```
pepper_variant call_variant -b $BAM -f $REF -o $ROOT/pepper -m $PEPPER_MODEL -t $THREADS
-s Sample --ont_r10_q20 --include-supplementary --no_quantized 2>&1 |tee $ROOT/pepper/pepper.log
```

5. Benchmarking vcf using hap.py

```
python hap.py $GIAB_BASELINE_VCF PEPPER_VARIANT_FULL.vcf.gz -o $OUT_DIR/happy -r $REF --threads
$THREADS --pass-only --engine=vcfeval
```

6. Running Margin(pepper_deepvariant_r0.8)

```
singularity run $PEPPER_MARGIN_SIF margin phase $BAM $REF $INPUT_VCF $PARAM_FILE -t $THREADS
--skipHaplotypeBAM -o $OUT_DIR/MARGIN_PHASED 2>&1 |tee $OUT_DIR/margin.log
```

GIAB truth vcf can be found in HG002 and HG001

7. Running Whatshap(v1.7)

```
whatshap phase --ignore-read-groups --reference $REF -o $OUT_DIR/phased.vcf $INPUT_VCF $BAM
```

8. Running HapCUT2(v1.3)

```
extractHAIRS --indels $USE_INDELs --ont 1 --bam $BAM --VCF $INPUT_VCF --ref $REF
--out $OUT_DIR/fragment_file
HAPCUT2 --outvcf 1 --fragments $OUT_DIR/fragment_file --VCF $INPUT_VCF
--output $OUT_DIR/haplotype_output_file
```

9. Benchmarking using whatshap(v1.7)

```
whatshap stats --only-snvs $OUT_DIR/MARGIN_PHASED.phased.vcf > $OUT_DIR/stats.log
whatshap compare --only-snvs --ignore-sample-name --tsv-pairwise $OUT_DIR/compare.tsv
$OUT_DIR/MARGIN_PHASED.phased.vcf $TRUTH_VCF > $OUT_DIR/compare.log
```

10. Generating reference assembly using Flye(v2.9.4-b1799)

```
flye --threads $THREADS --nano-hq all.fastq -o $OUT_DIR/flye_hap
```

11. Running Hapdup-NanoFilter

```
$HAPDUP_ROOT/hapdup.py --use-multiprocess --$MODE --assembly $DATA_DIR/flye_hap/assembly.fasta
--bam $DATA_DIR/all_assembly.bam --out-dir $OUT_DIR -t $THREADS --rtype $DATA_TYPE
```

12. Benchmarking assemblies using Merqury(v1.3)

```
merqury.sh $MERYL_ROOT/hg002_k21.meryl $MERYL_ROOT/hg004_k21.hapmer.meryl
$MERYL_ROOT/hg003_k21.hapmer.meryl $HAPDUP_ROOT/hapdup_mode_1.fasta $HAPDUP_ROOT/hapdup_mode_2.fasta
bench
```

Available pre-built meryl dbs can be found in MERYL_DB

13. Benchmarking assemblies using Quast(v5.2.0)

```
quast.py $ROOT/$HAPDUP_DIR/hapdup_phased_merged.fasta -o $ROOT/$HAPDUP_DIR/quast_results
-t 40 -r $HG002_REF
```

HG002 Reference is available in HG002 T2T Reference

2 Filtering parameter selection

Table S1: Filtering performance under different (p1, p2, p3) combinations. p1, p2, and p3 correspond to the first, second, and third filtering thresholds applied in the three-step filtering strategy

**p1**	**p2**	**p3**	**N50**	**switch errors count**
0.60	0.70	0.80	972361	364
0.60	0.70	0.85	966451	363
0.60	0.70	0.90	964409	360
0.60	0.70	0.95	956746	370
0.60	0.75	0.80	972361	365
0.60	0.75	0.85	966451	361
0.60	0.75	0.90	964409	362
0.60	0.75	0.95	956746	370
0.60	0.80	0.85	966451	361
0.60	0.80	0.90	964409	359
0.60	0.80	0.95	956746	370
0.60	0.85	0.90	964409	362
0.60	0.85	0.95	956746	370
0.65	0.70	0.80	967932	360
0.65	0.70	0.85	964954	357
0.65	0.70	0.90	963779	356
0.65	0.70	0.95	955271	365
0.65	0.75	0.80	967932	359
0.65	0.75	0.85	964954	357
0.65	0.75	0.90	963779	356
0.65	0.75	0.95	955271	367
0.65	0.80	0.85	964954	356
0.65	0.80	0.90	963779	355
0.65	0.80	0.95	955271	365
0.65	0.85	0.90	963779	354
0.65	0.85	0.95	955271	364
0.70	0.75	0.80	966451	358
0.70	0.75	0.85	964954	354
0.70	0.75	0.90	963167	352
0.70	0.75	0.95	954802	361
0.70	0.80	0.85	964954	353
**0.70**	**0.80**	**0.90**	**963167**	**351**
0.70	0.80	0.95	954802	361
0.70	0.85	0.90	963167	353
0.70	0.85	0.95	954802	360
0.75	0.80	0.85	964082	354
0.75	0.80	0.90	962759	353
0.75	0.80	0.95	954241	362
0.75	0.85	0.90	962759	353
0.75	0.85	0.95	954241	361

3 Haplotagging accuracy evaluation

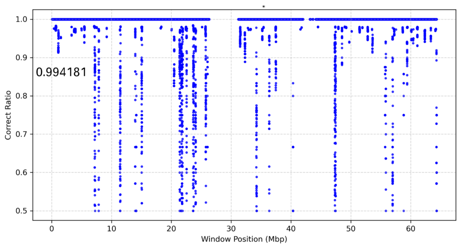

(a) SNVs only

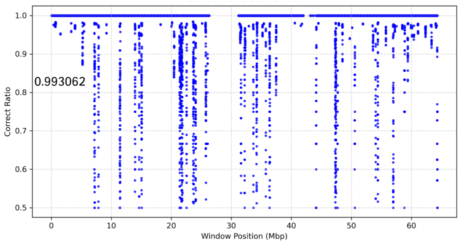

(b) SNVs with raw INDELs

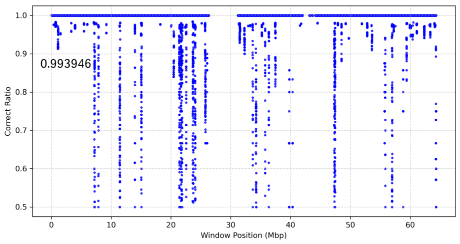

(c) SNVs with INDELs filtered by NanoFilter

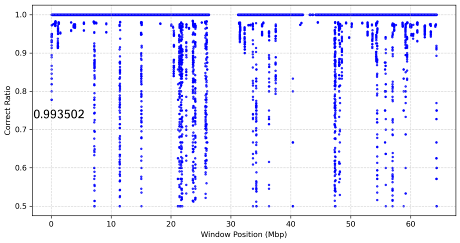

(d) SNVs with INDELs filtered by quality 15

Figure S1: Comparison of haplotagging accuracy with different phased variant sets.

4 Visualization of Assembly Results

To evaluate the performance of NanoFilter on haplotype-resolved assembly, we used the HG002 T2T v1.1 reference genome (released in 2024, `https://github.com/marbl/HG002`) as the ground truth for parental origin and as a benchmark for detecting misassemblies. We developed a custom script to compare QUAST outputs across assembly results and identify regions where misassemblies are improved. Below, we present representative regions that highlight the impact of INDEL and NanoFilter on assembly process.

4.1 R9 chr4_MATERNAL / contig_2840

As shown in Figure S2, assembly result based solely on raw SNVs introduces misassemblies in contig_2840. Incorporating filtered INDELs corrects this misassembly.

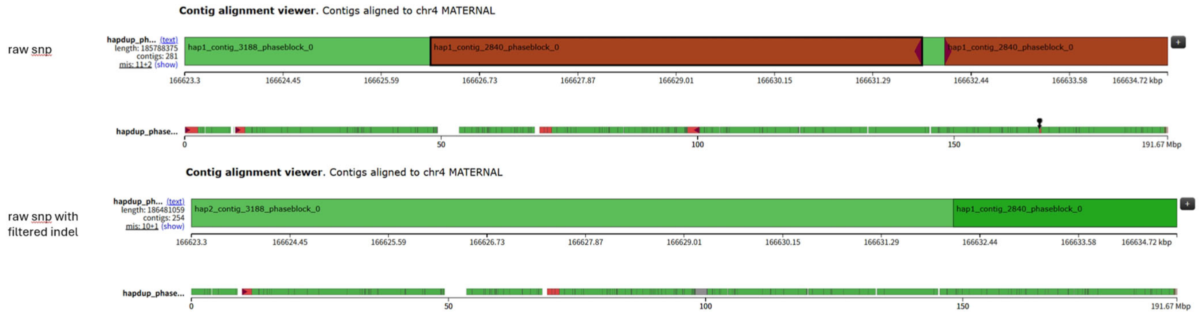

Figure S2: Correction of local misassemblies in contig_2840 through the inclusion of filtered INDELs.

Figure S3 shows that INDELs filtered by NanoFilter enable consistent alignment of maternal reads (HP=1) across the region. In contrast, phasing based on SNVs causes a haplotype switch at the rightmost segment, leading to erroneous paternal read incorporation and misassembly.

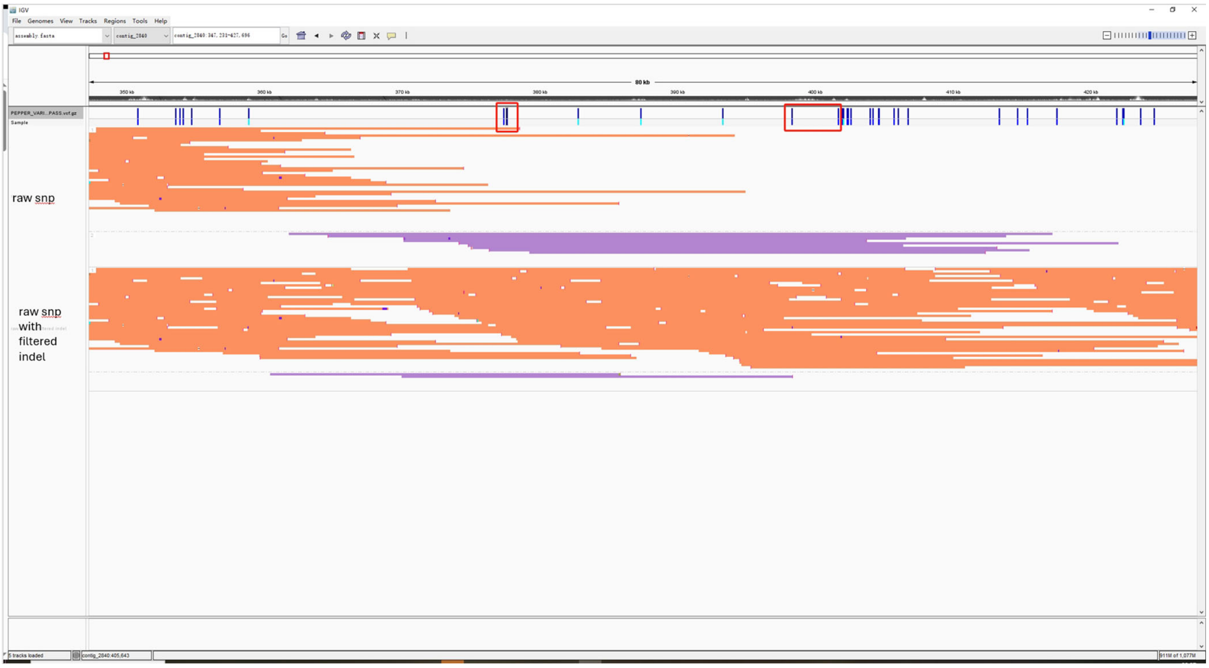

Figure S3: Haplotype-resolved alignment on contig_2840 showing improved phasing and assembly with filtered INDELs. Reads are color-coded based on parental origin.

4.2 R9 chr9_PATERNAL / contig_470

As shown in Figure S4, both raw SNVs phasing and raw SNVs with raw INDELs phasing produce misassemblies on contig_470. Using raw SNVs and filtered INDELs resolves assembly result this region.

Figure S5 shows that raw SNVs phasing fails to exclude maternal reads, while raw INDELs introduce additional noise. After filtering two INDELs in this region, clear phasing boundaries form, enabling correct haplotype assignment.

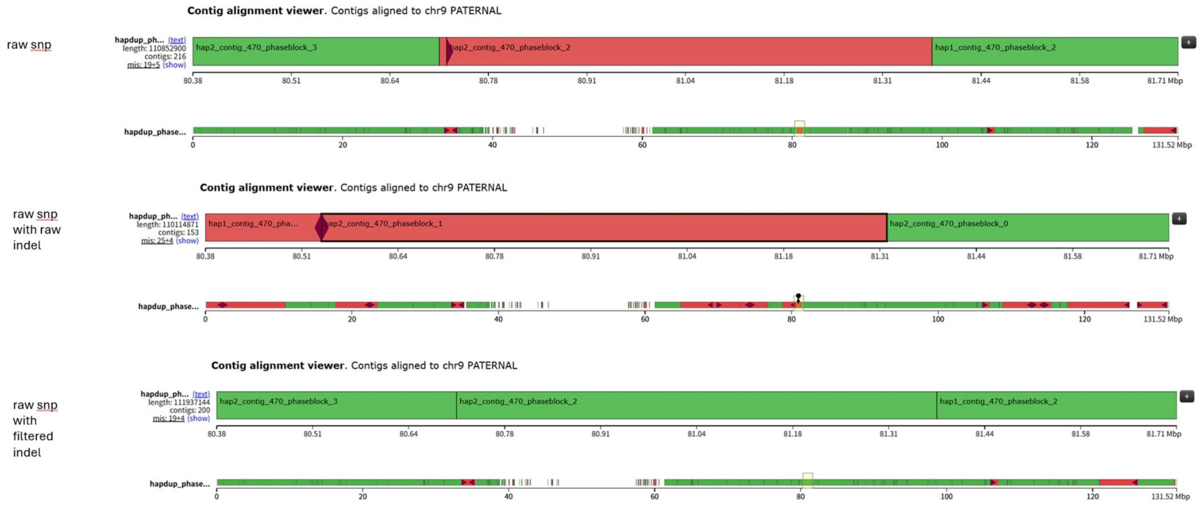

Figure S4: Improved assembly of contig_470 after filtering out inconsistent INDELs. Misassemblies from unfiltered variants are eliminated.

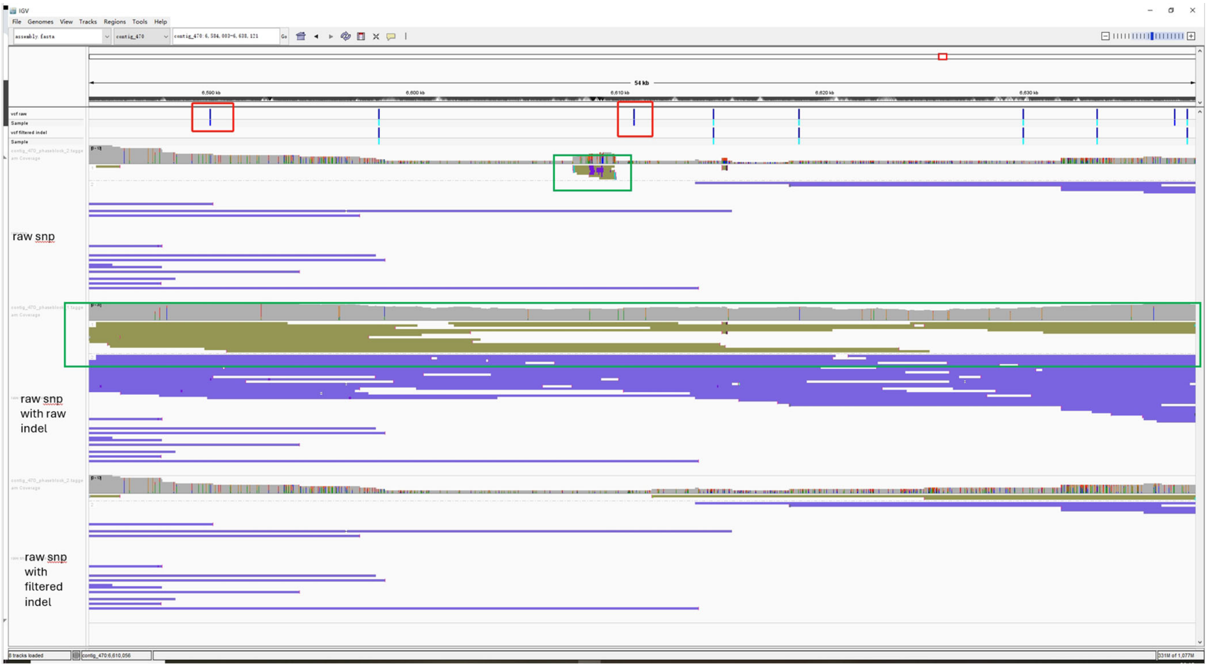

Figure S5: Haplotype-specific read alignment on chr9_PATERNAL and contig_470 after filtering. Blue reads represent correctly phased paternal reads.

4.3 R9 chr15_MATERNAL / contig_470

Figure S6 demonstrates that NanoFilter is critical for eliminating inconsistent INDELs and achieving accurate assembly in this region.

Figure S7 shows that misassigned reads and a haplotype switch when raw SNVs and raw INDELs are used. After filtering low-consistency INDELs, the correct maternal haplotype is restored, improving phasing performance and assembly contiguity.

As shown in Figure S8, three low-consistency INDELs (highlighted in red blocks) are removed to purify the middle segment. Remaining high-confidence INDELs aid in bridging to adjacent reads.

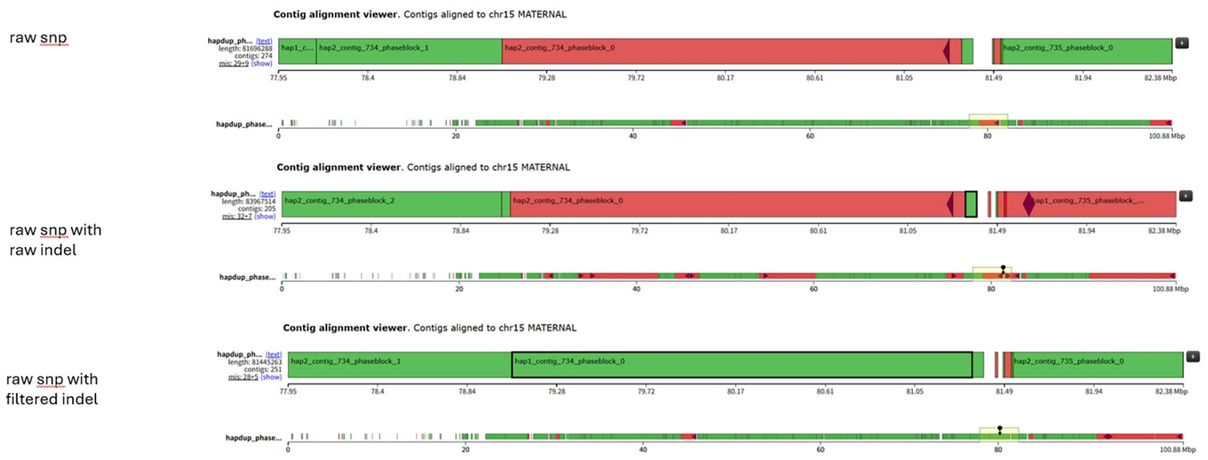

Figure S6: Assembly accuracy improvement on chr15_MATERNAL after NanoFilter removes erroneous INDELs.

4.4 R10 simplex chr1_MATERNAL / contig_1254

As shown in Figure S9, the region highlighted in red block contains only SNVs, and incorporating these raw SNVs alone into the assembly process results in misassemblies. However, the presence of two flanking INDELs (TTG/T and C/CT) provides critical phasing information that enables the correct haplotype reconstruction. Including these INDELs substantially improves phasing accuracy and enables correct haplotype assignment across the region.

4.5 R10 simplex chr10_PATERNAL / contig_1646

Figure S10 highlights a translocation error caused by phasing with raw SNVs. The left segment is misassembled under chr10_MATERNAL. Adding two informative INDELs (TA/T and GA/G) corrects phasing and ensures proper assignment to chr10_PATERNAL.

4.6 R10 simplex contig_2689

Figure S11 illustrates a case where the inclusion of INDELs filtered by NanoFilter, resulted in worse assembly performance compared to using SNVs alone. Although a short misassembly is observed in the assembly result of raw SNVs, reads soon revert to the correct haplotype, avoiding translocations. However, after adding INDELs, reads switch to the alternate haplotype, producing a translocation.

Figure S12 shows the variant distribution across the 73 kbp misassembled region. Only three SNVs and two INDELs are present, limiting NanoFilter's ability to filter variants effectively. The retained INDELs misguide phasing, leading to erroneous read assignment and misassemblies.

4.7 Analysis of optimized regions

The method for identifying optimized regions between assembly results is as follows. For any phaseblock in an assembly that contains no misassemblies, we compare the corresponding regions in other assemblies to detect cases where these alternative assemblies exhibit misassemblies. Each identified misassembly is extended by 50 kbp upstream and downstream to define a broader misassembly region. Within these regions, we calculate the density of heterozygous raw SNVs and INDELs. In the following plots, "rs", "rsri", "rsfi", and "fsfi" refer to assemblies constructed using raw SNVs only, raw SNVs combined with raw INDELs, raw SNVs combined with INDELs filtered by NanoFilter, and SNVs and INDELs filtered by NanoFilter, respectively.

For the R9 dataset (Figure S13), the rs assembly produces more optimized regions than the other three combinations, with 180, 83, and 90 optimized regions, respectively. In contrast, rsfi and fsfi outperform rs in only 76 and 61 regions, respectively. Notably, in regions where rsri and fsfi outperform rs, the density of SNVs and INDELs were markedly higher.

For the R10 simplex dataset (Figure S14), rs outperforms the other combinations in 225, 239, and 206 regions, respectively, while rsfi alone outperforms rs in 233 regions. only slightly fewer than rs's

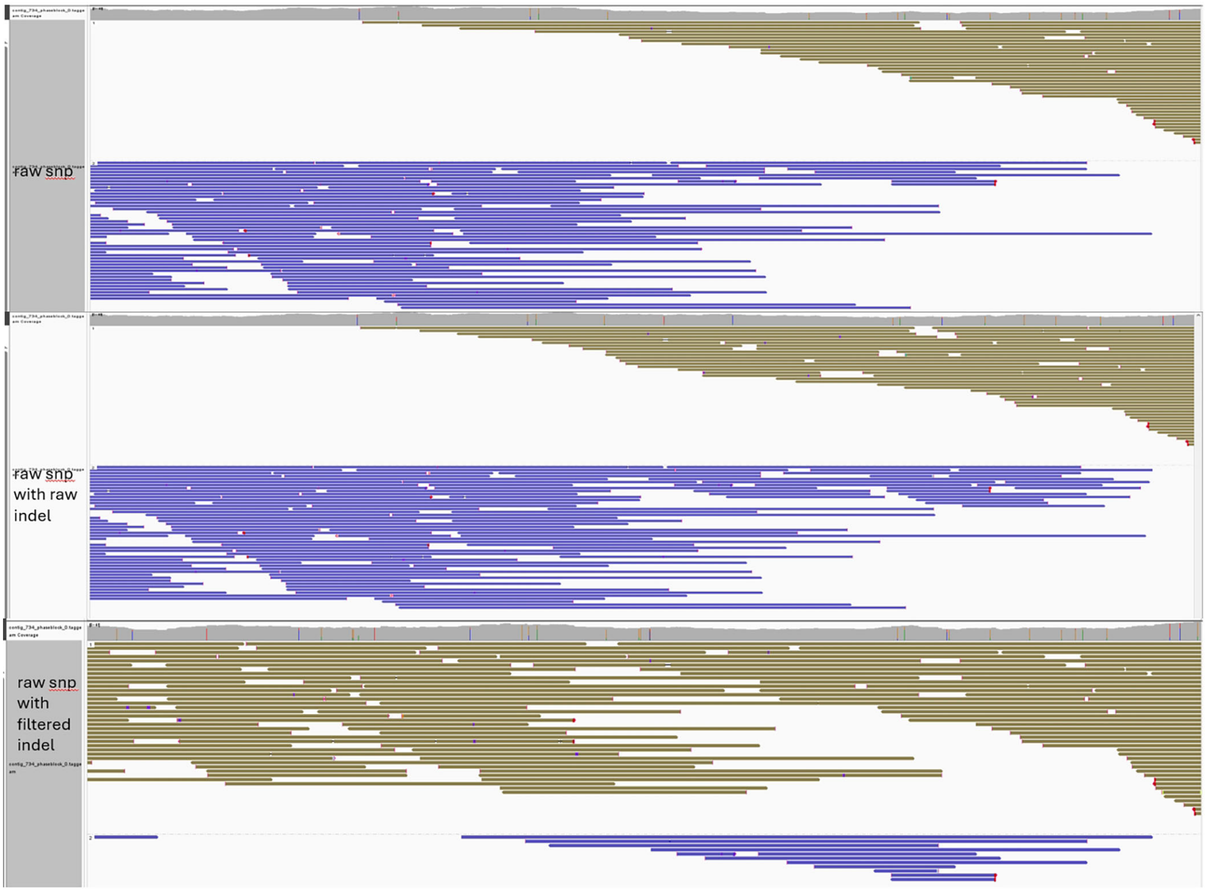

Figure S7: Read alignment on chr15_MATERNAL and contig_470 showing improved haplotype assignment after filtering. Green reads represent maternal origin.

advantage over rsfi. The fsfi combination outperforms rs in 255 regions. In all combinations involving INDELs, regions that outperformed rs consistently showed higher SNV and INDEL density.

Similarly, in the R10 duplex dataset (Figure S15), rs outperforms the other combinations in 182, 173, and 186 regions, while rsfi and fsfi outperformed rs in 187 and 195 regions, respectively. Again, regions that benefited from INDEL incorporation exhibited elevated INDEL densities.

Collectively, these results suggest that NanoFilter, leveraging variant consistency to assess phasing reliability, performs best in regions with abundant heterozygous SNVs and INDELs. This facilitates more accurate filtering of variants and leads to improved assembly outcomes.

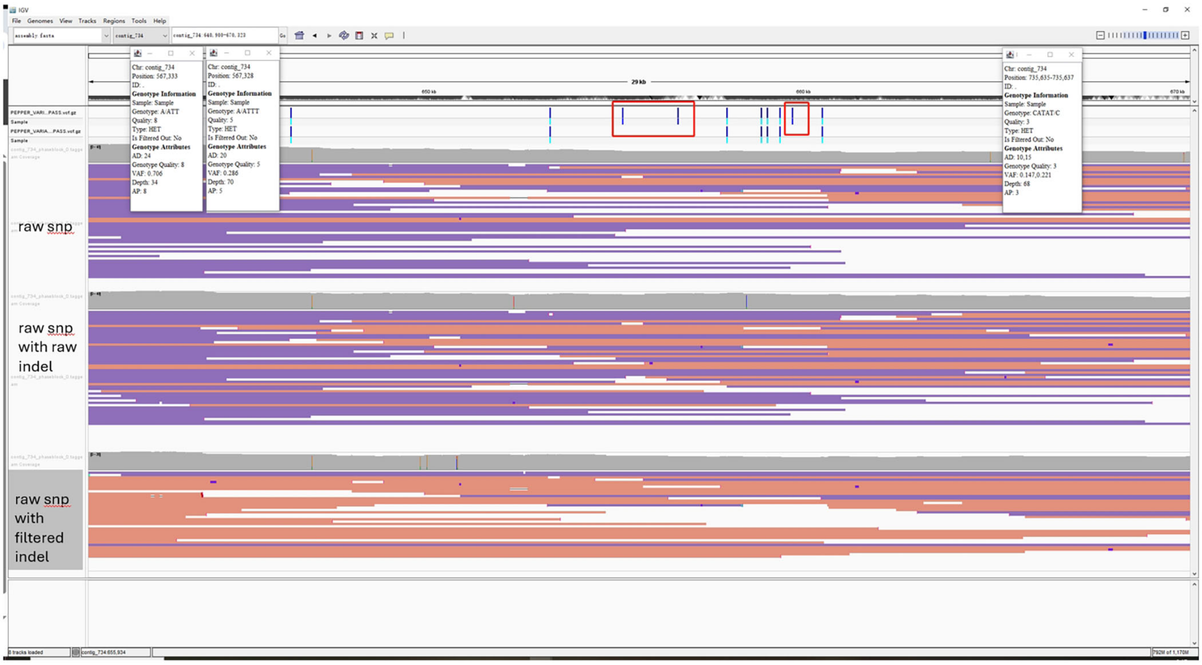

Figure S8: Filtered variant distribution on chr15_MATERNAL and contig_470 showing removal of erroneous INDELs and retention of informative boundary variants.

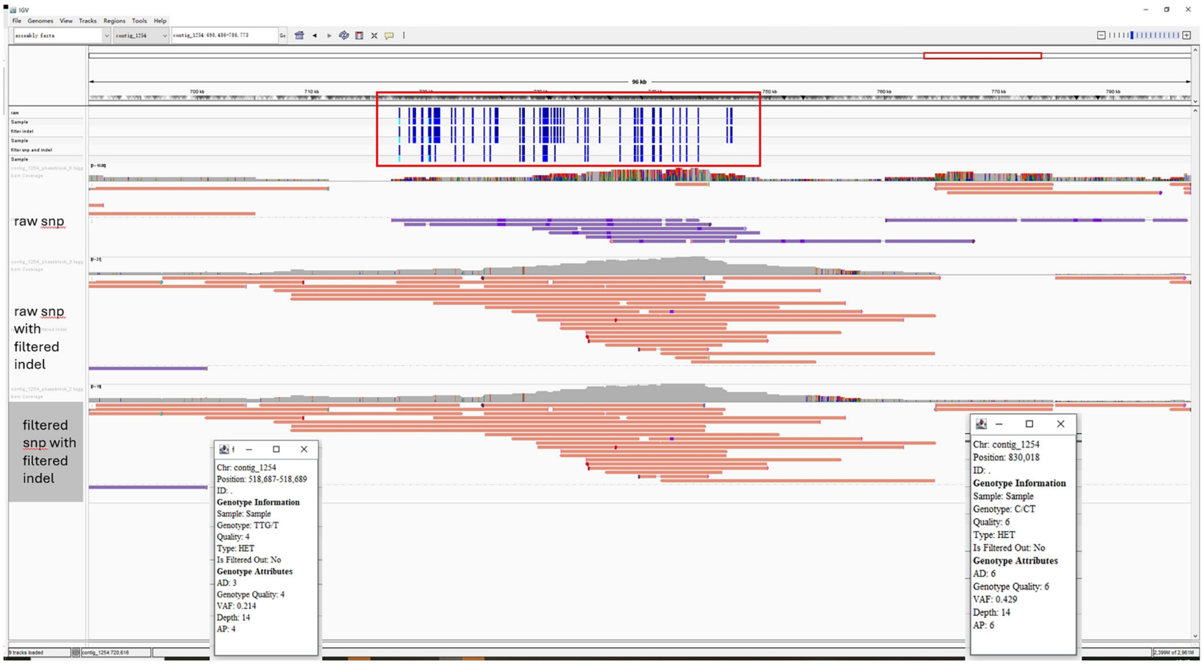

Figure S9: Improved haplotype phasing on chr1_MATERNAL enabled by informative flanking INDELs in a region containing only SNVs.

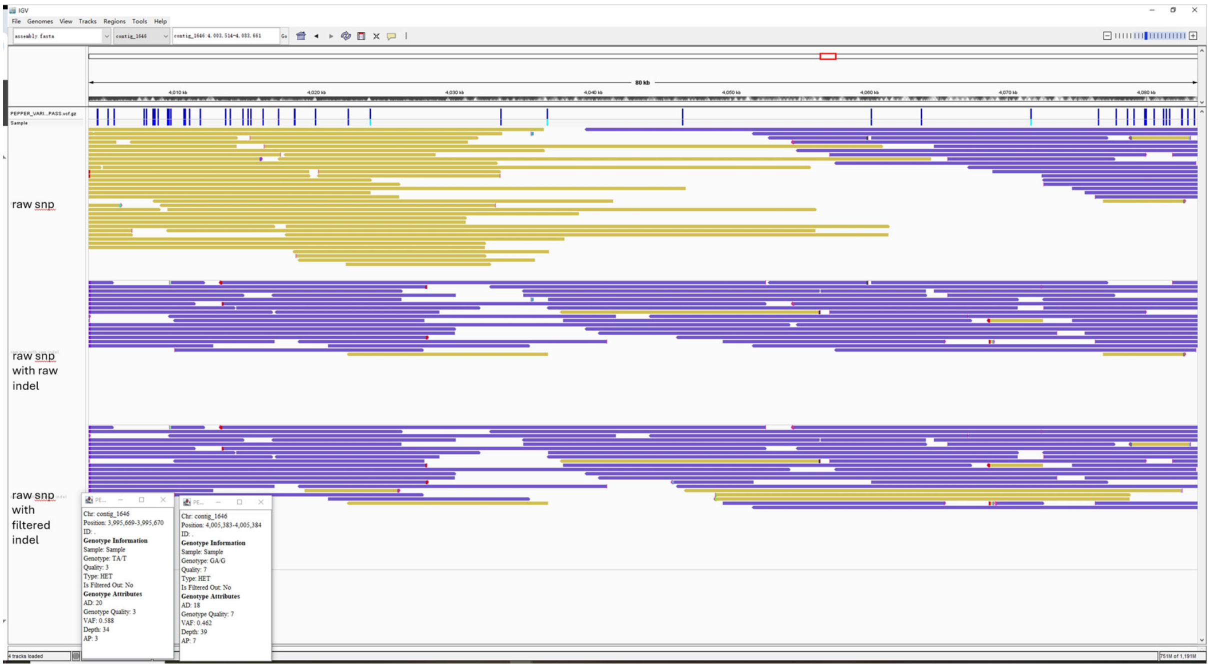

Figure S10: Correction of a translocation on chr10_PATERNAL enabled by phasing with informative INDELs.

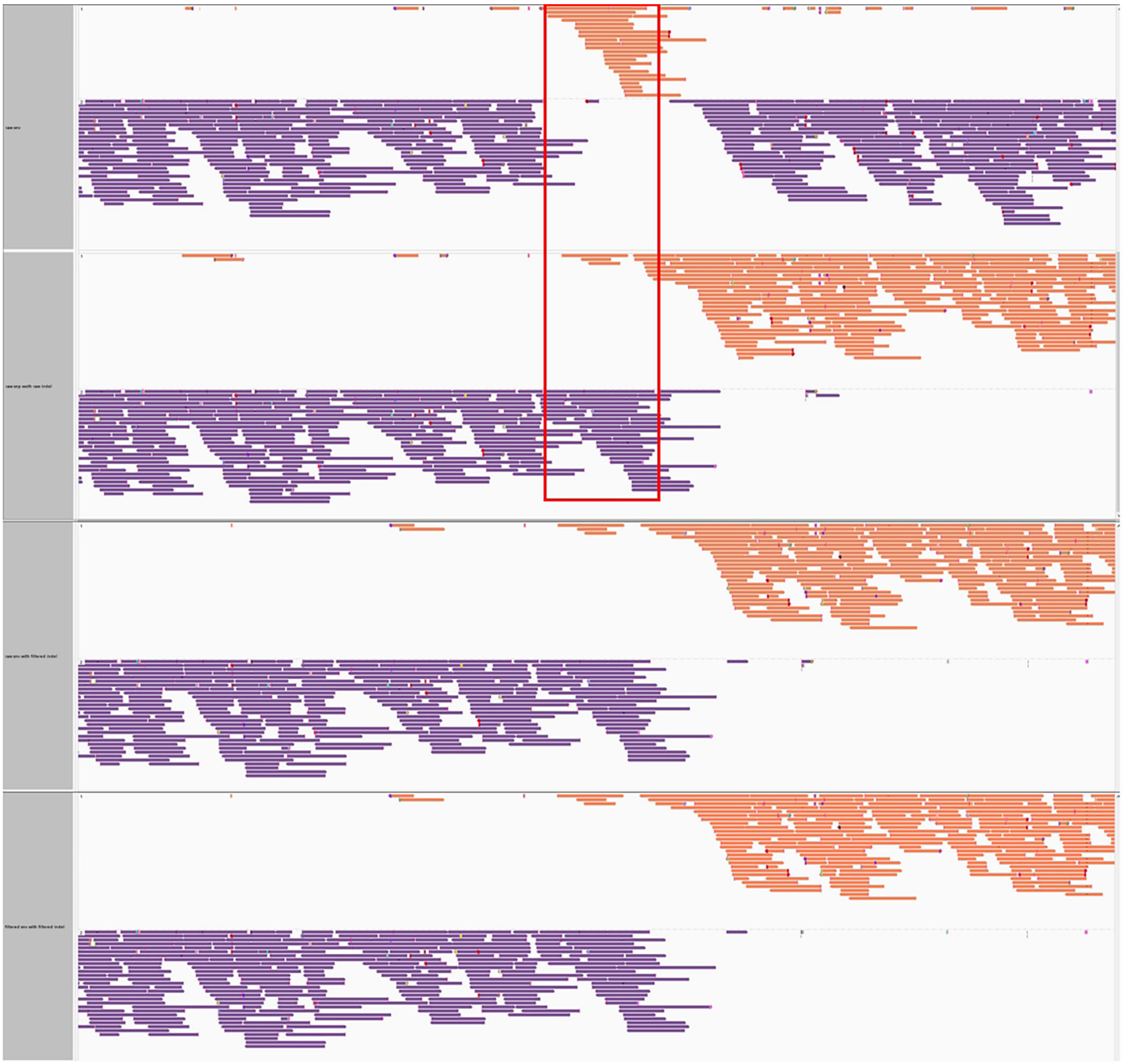

Figure S11: Incorrect haplotype switch and translocation caused by sparse and misfiltered INDELs in contig_2689.

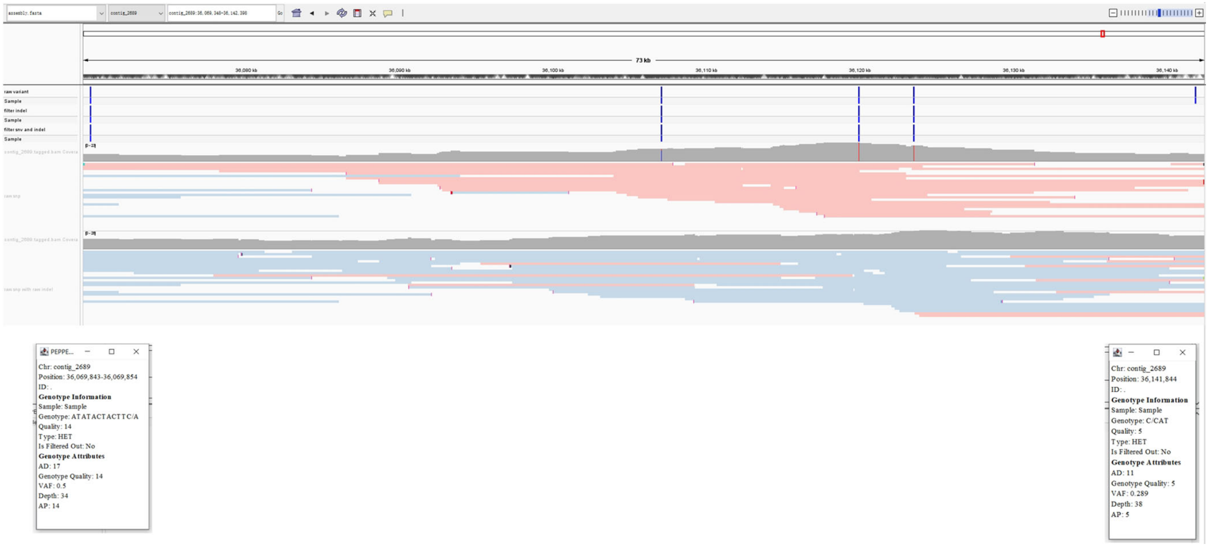

Figure S12: Sparse variant landscape in the misassembled region of contig_2689, with only three central SNVs and flanking INDELs. Lack of informative variants led to incorrect haplotype switching.

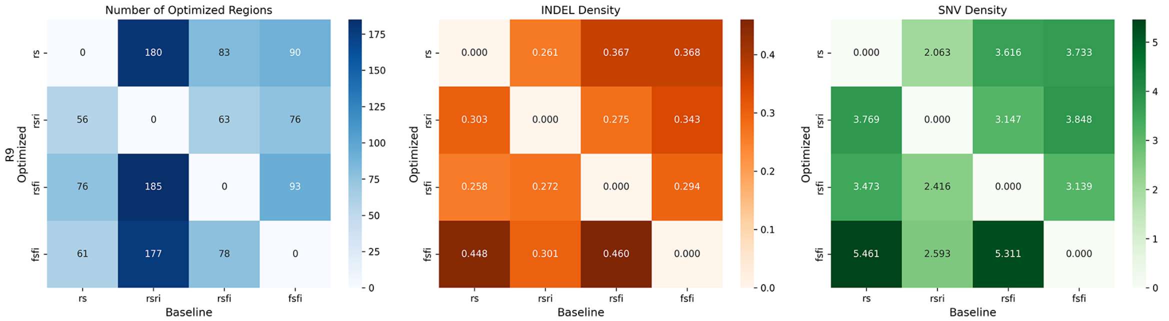

Figure S13: Comparison of optimized regions, local INDEL density, and SNV density across variant set transitions in R9 data.

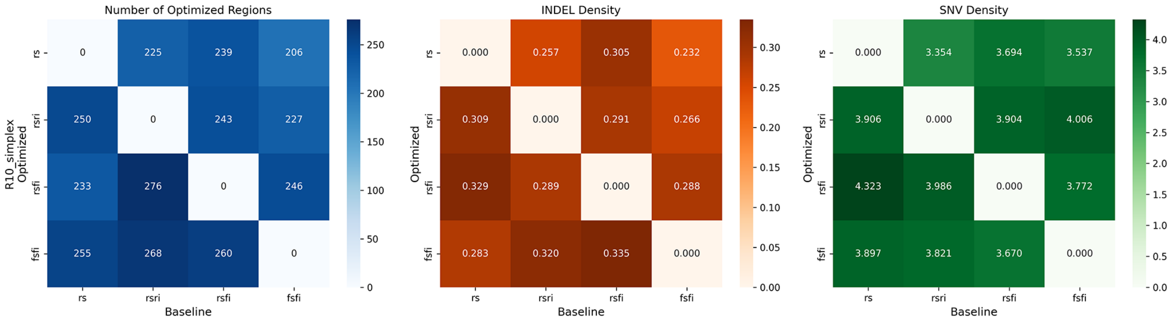

Figure S14: Comparison of optimized regions, local INDEL density, and SNV density across variant set transitions in R10 simplex data.

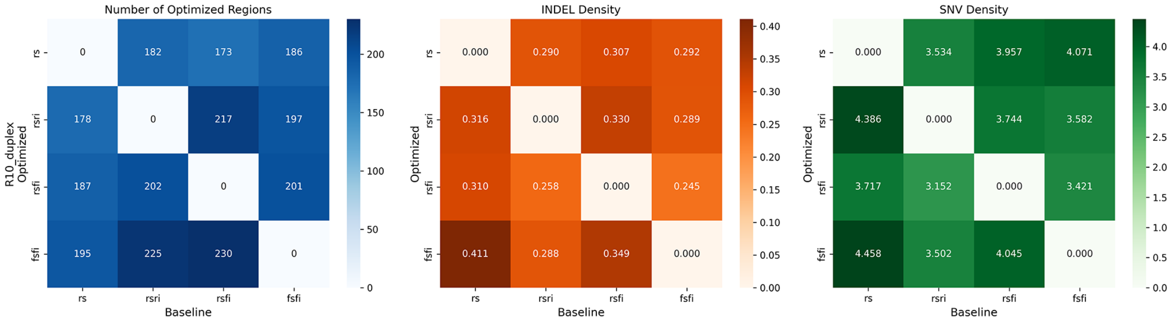

Figure S15: Comparison of optimized regions, local INDEL density, and SNV density across variant set transitions in R10 duplex data.
